# Perioperative Management Challenges in Silent Pheochromocytoma: A Case Report and Literature Review

**DOI:** 10.1002/ccr3.70396

**Published:** 2025-04-06

**Authors:** Kai Lin, Xuan Li, Tian Guo, Haixing Zhong

**Affiliations:** ^1^ Department of Anesthesiology and Perioperative Medicine, Xijing Hospital The Fourth Military Medical University Xi'an China; ^2^ Shaanxi Provincial Clinical Research Center for Anesthesiology Medicine Xi'an China; ^3^ Xi'an Medical University Xi'an China; ^4^ Medical College of Yan'an University Yan'an China

**Keywords:** adrenal incidentaloma, case report, hypertensive crisis, perioperative management, silent pheochromocytoma

## Abstract

Silent pheochromocytomas, frequently misdiagnosed as nonfunctioning adrenal adenomas, carry a high risk of perioperative hemodynamic instability. This case underscores the importance of thorough preoperative biochemical screening, proactive anesthetic strategies, and continuous intraoperative monitoring. Effective multidisciplinary management is crucial to prevent life‐threatening complications and achieve optimal surgical outcomes.

## 1 Introduction

Arising from chromaffin cells, pheochromocytoma (PCC, accounting for 85%–90%) and paraganglioma (PGL, accounting for 10%–15%) (collectively referred to as PPGL) are characterized by the excessive secretion of catecholamines (CA), leading to paroxysmal or persistent hypertension accompanied by headache, diaphoresis, and palpitations [1, 2]. The prevalence of PPGL is approximately 0.57 cases per 100,000 person annually [3]. Among them, around 3%–10% of patients present with an absence of characteristic clinical features and are often misdiagnosed as nonfunctioning adrenal adenomas referred to as “silent” PCCs. Failure to recognize this preoperatively may lead to significant intraoperative hemodynamic instability with potentially life‐threatening complications, such as hypertensive crises or heart failure [4]. Preoperative thorough preparation significantly reduces perioperative mortality in PCC patients to nearly 0%, whereas insufficient preparation can result in mortality rates as high as 43% [5]. Therefore, the perioperative management of silent PCCs holds critical clinical importance, while posing challenges for anesthesiologists.

In this report, we present a case of silent PCC along with its perioperative management and review the relevant literature, aiming to enhance understanding, management strategies, and safety for this critical patient.

## 2 Case Presentation

### 2.1 Case History

**FIGURE 1:**
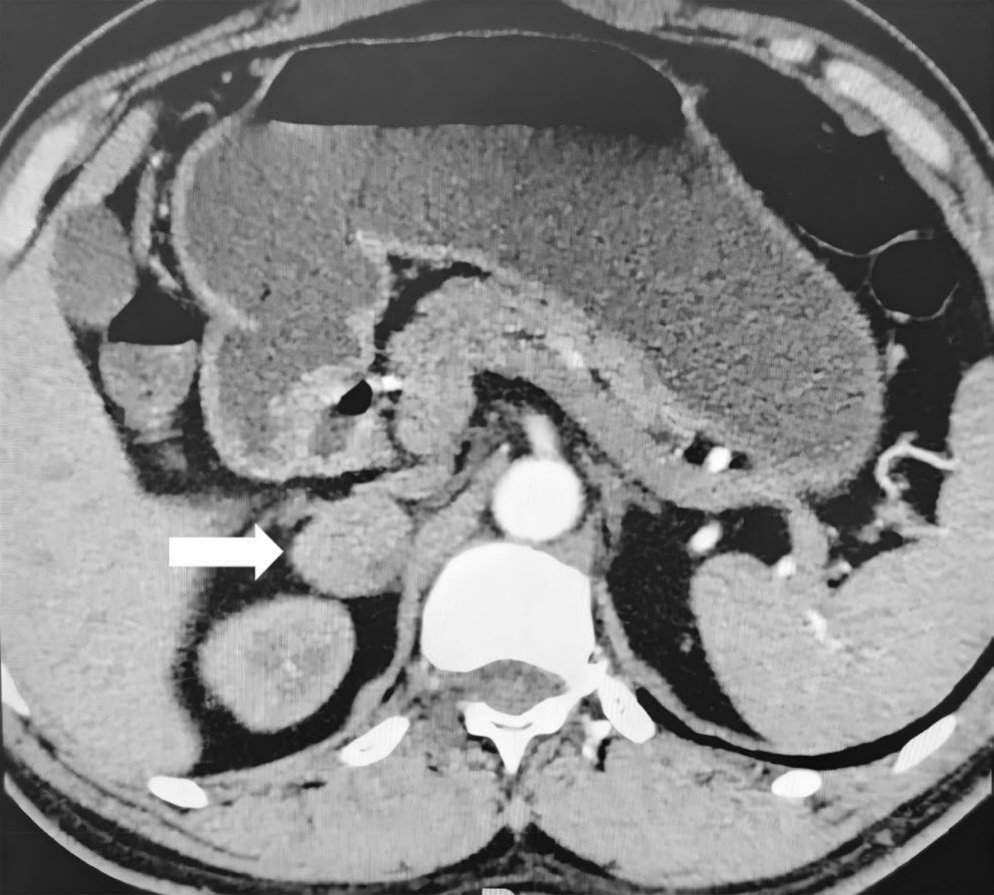
The axial CT image reveals a clearly demarcated mass (indicated by the white arrow) located in the right adrenal gland region.

The patient, a 50‐year‐old male with a height of 170 cm and weight of 69 kg, was admitted to the hospital due to an incidentally detected right adrenal mass during a routine health examination. Enhanced adrenal CT imaging revealed a well‐defined heterogeneous mass measuring approximately 2.8 × 2.2 cm in the right adrenal gland (Figure 1). Laboratory results, including six tests for hypertension (fasting standing position), showed the following: Adrenal‐related markers: adrenocorticotropic hormone (ACTH) 20.37 pg/mL, renin 26.40 uIU/mL, aldosterone‐to‐renin ratio (ARR) 2.69, aldosterone (Aldo) 71.0 pg/mL, cortisol (Cor) 7.49 μg/dL, and angiotensin II (AII) 36.90 pg/mL. Secondary hypertension markers: vanillylmandelic acid (VMA) 10.5 mg/24 h, 17‐hydroxycorticosteroids (17‐OH) 2.2 mg/24 h, 17‐ketosteroids (17‐KS) 5.7 mg/24 h, dehydroepiandrosterone sulfate (DHEA‐S) 0.6 mg/24 h, and free cortisol (Cor) 189.3 μg/24 h—all within normal ranges. No abnormalities were found in other laboratory tests. Additionally, The patient denied experiencing sudden dizziness, headaches, palpitations, diaphoresis, nausea, or vomiting episodes, as well as hypertension or having any relevant medical or family history.

### 2.2 Differential Diagnosis, Investigations and Treatment

Based on the patient's medical history and other relevant examinations, the surgeon established a definitive diagnosis of a nonfunctioning adrenal adenoma without further differential diagnosis. Subsequently, the patient was scheduled to undergo a laparoscopic right adrenalectomy under general anesthesia. For preoperative preparation, a daily infusion of 3000 mL of intravenous fluids was administered over 3 days to expand the volume, with a colloid‐to‐crystalloid solution ratio of 1:2.

Upon arrival in the operating room, the patient was adequately fasted and comfortable. Routine monitoring including electrocardiography, invasive blood pressure measurement, pulse oximetry, and bispectral index (BIS) was performed, and the baseline vital signs were within normal range. Anesthetic induction was performed using 20 mg of propofol, 30 μg of sufentanil, and 50 mg of rocuronium. Following tracheal intubation, mechanical ventilation was initiated with a tidal volume of 500 mL, a respiratory rate of 12 breaths per minute (bpm), an inspiratory‐to‐expiratory ratio of 1:1.5, and a positive end‐expiratory pressure (PEEP) set at 6 mmHg to maintain the pressure of end‐tidal CO_2_ (PET CO_2_) levels between 35 and 45 mmHg. Anesthesia was maintained with sevoflurane at concentrations ranging from 1% to 2%, along with continuous intravenous infusions of dexmedetomidine (0.4 μg/kg/h) and remifentanil (0.15 μg/kg/min), targeting a BIS range of 40–60. Rocuronium administration was adjusted as necessary to ensure adequate muscle relaxation. The patient was positioned in the left lateral decubitus position with a pneumoperitoneum pressure of 12 mmHg.

**FIGURE 2:**
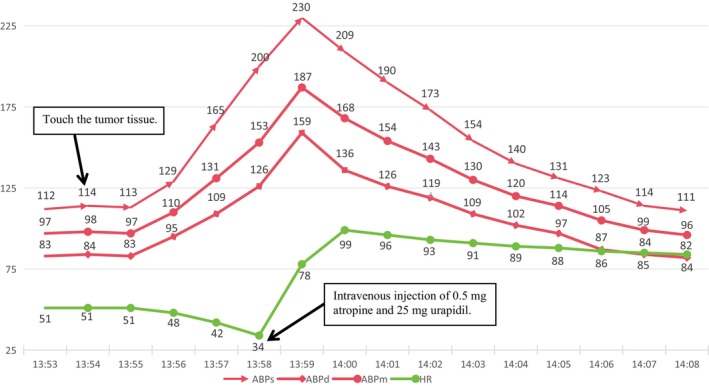
Hemodynamic changes (ABPs, ABPd, ABPm, and HR) over time during tumor stimulation. *X*‐axis: Time (minutes). *Y*‐axis: Hemodynamic parameters (mmHg for blood pressure, bpm for heart rate).

**FIGURE 3:**
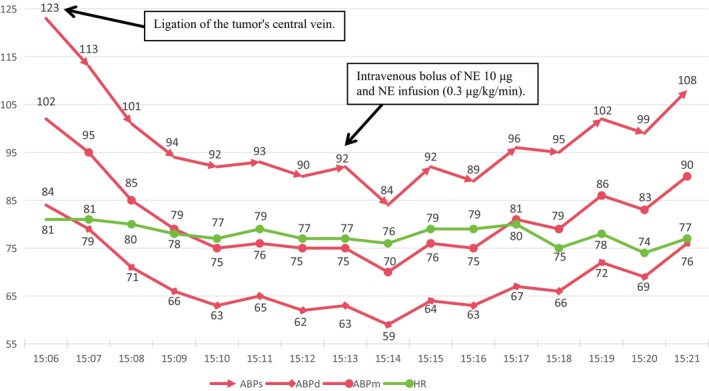
Hemodynamic changes (ABPs, ABPd, ABPm, and HR) over time after central venous ligation. *X*‐axis: Time (minutes). *Y*‐axis: Hemodynamic parameters (mmHg for blood pressure, bpm for heart rate).

The vital signs kept stable until the exposure and stab of the yellow, well‐demarcated mass at the upper pole of the right kidney. There was a sudden surge in blood pressure to 230/159 mmHg accompanied by a drop in heart rate to 34 bpm. Subsequently, the surgical procedure was immediately suspended, and prompt administration of 0.5 mg atropine and 25 mg urapidil ensued. As a result, hemodynamic stability was restored with a heart rate of 85 bpm and blood pressure of 114/84 mmHg (Figure 2), resulting in a possible revised diagnosis of norepinephrine (NE)‐secreting PCC. To achieve further volume expansion, intravenous fluid administration was accelerated using crystalloid and colloid solutions. Five minutes after achieving hemodynamic stabilization, surgery resumed under close monitoring with intermittent hypertension. Boluses of nicardipine (0.2–0.4 mg/dose) were administered to maintain blood pressure within the range of 110–140/80–100 mmHg, while 1–2 mg/dose esmolol was given to control the heart rate at 70–90 bpm. The nourishing arteries of the tumor were gradually ligated, and finally, when the central vein was ligated, the patient experienced a sudden decrease in blood pressure, reaching a nadir of 84/59 mmHg. NE was intermittently administered in boluses of 10 μg, followed by continuous infusion at 0.3 μg/kg/min until the blood pressure stabilized at approximately 110/80 mmHg, with a heart rate around 75 bpm (Figure 3). Afterward, bolus administration was stopped, and the infusion rate of NE was gradually reduced for about 40 min when blood pressure was stabilized at approximately 115/80 mmHg and heart rate around 75 bpm. During this period, the tumor and part of the adrenal gland were also completely resected. The surgery persisted for 260 min without complications. Estimated blood loss was 20 mL, urine output was 500 mL, and a total of 3000 mL of fluids (2000 mL Ringer's solution, 1000 mL hydroxyethyl starch sodium chloride solution) was administered intraoperatively. Perioperative arterial blood gas results—including preoperative, during tumor manipulation, post‐tumor resection, and upon admission to the postanesthesia care unit—were consistent with typical perioperative arterial blood gas changes, with no significant abnormalities observed (Table 1).

**TABLE 1:** Perioperative changes in arterial blood gas of the patient.

aBGA—timing	Preoperative (12:30)	During tumor manipulation (14:05)	Post‐tumor resection (15:24)	Postanesthesia care unit (16:55)
PH	7.41	7.31	7.3	7.4
PCO_2_ (mmHg)	39	46	46	38
PO_2_ (mmHg)	205	166	182	202
Na^+^ (mmol/L)	136	135	137	138
K^+^ (mmol/L)	3.3	4.3	3.7	3.6
Ca^+^ (mmol/L)	1.12	1.1	1.06	1.09
Glu (mmol/L)	5.8	8.3	10.3	10.5
Lac (mmol/L)	1.1	0.7	2.2	1.7
HCt (%)	34	34	35	33
FIO_2_ (%)	55	55	55	50
${\mathrm{HCO}}_{3}^{-}$ (mmol/L)	25	22.3	21.8	22.2
BE (mmol/L)	0.1	−3.3	−4	−3.5
SO_2_c (%)	99.4	99.4	97.7	98.9
tHb (g/dL)	13.5	13.2	13.4	13.3

### 2.3 Outcome and Follow‐Up

**FIGURE 4:**
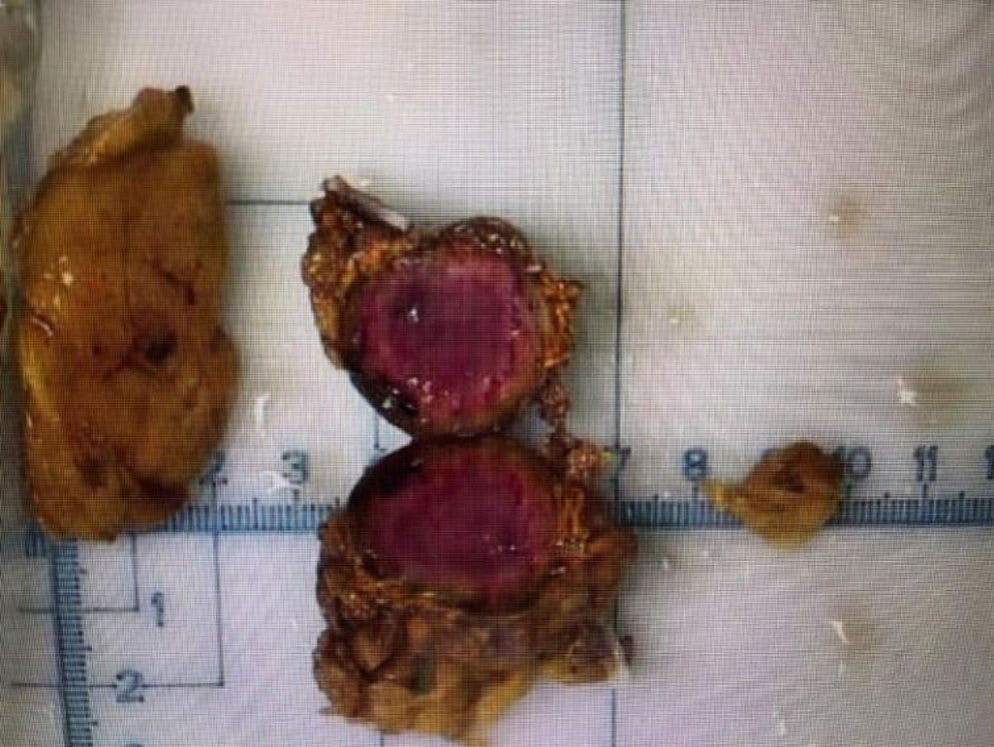
Gross morphology of the excised tumor. The excised tissue, measuring 3.5 cm × 3.5 cm × 2.8 cm, is an irregular gray‐yellow mass weighing 16.2 g. The cut surface reveals a central, well‐demarcated, gray‐red tumor measuring 2.3 cm × 2.0 cm.

**FIGURE 5:**
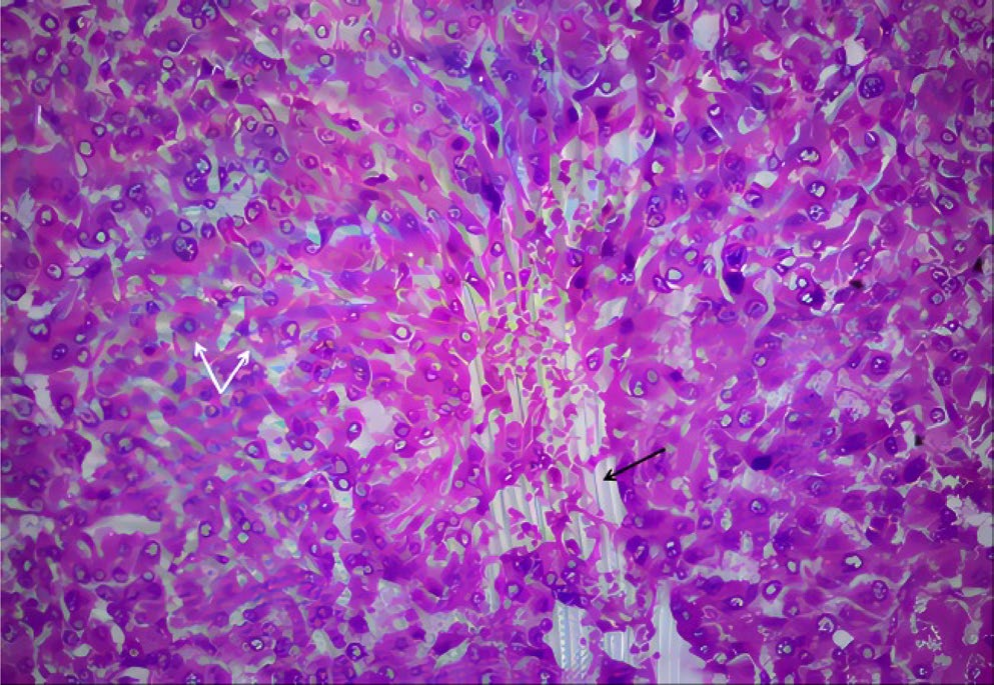
HE staining of the tumor tissue (magnification, ×40). Immunohistochemical analysis reveals polygonal or round tumor cells indicated by white arrows, characterized by abundant foamy and eosinophilic cytoplasm. The black arrows point to scattered capillaries within the tumor tissue.

Postoperatively, The gross morphology of the tumor is shown in Figure 4, and the histopathological features observed with HE staining are presented in Figure 5. Additionally, immunohistochemical analysis demonstrates that the tumor cells exhibit positive staining for chromogranin A (CgA), synaptophysin (Syn), CD56, S100, and Ki‐67 (hotspot area ~2%), while negative staining is observed for CYP11B1, CYP11B2, and SF‐1 markers. Reticulin special staining further reveals disrupted acinar architecture, supporting the diagnosis of a neuroendocrine neoplasm. The patient was discharged on the fifth postoperative day without any complications or discomfort. During the 6‐month follow‐up, the patient remained asymptomatic, with no evidence of recurrence or adverse events, and maintained overall good health. Further routine monitoring is recommended to ensure long‐term stability and to detect any potential late‐onset complications.

## 3 Discussion

Due to the increased frequency of routine physical examinations, the reported proportion of adrenal incidentaloma has risen from 31% during the period from 1995 to 2004 to 61% between 2005 and 2016 [6, 7]. Specifically, these adrenal incidentalomas are typically smaller in size (< 2 cm) and exhibit up to a median elevation in urinary metanephrine (MN) less than twice the upper limit of normal [8]. However, even mildly elevated MN levels can significantly increase the posttest probability of PCC by up to 80%, underscoring that small and indeterminate adrenal incidentalomas should not be disregarded as they may still represent potential PCCs [9].

In this specific case, despite undergoing routine volume expansion for three consecutive days prior to surgery, the patient diagnosed with a nonfunctioning adrenal adenoma still experienced significant hemodynamic fluctuations during the procedure. These fluctuations carried the potential to result in severe adverse outcomes, including stroke, hypertensive crisis, and cardiovascular emergencies [10]. Fortunately, due to meticulous preoperative preparation and effective intraoperative management, serious complications were successfully averted. Based on this case study, we have derived several key insights, as outlined below.

### 3.1 Preoperative Evaluation and Preparation

First, the clinical symptoms of patients with nonfunctioning adrenal adenoma necessitate heightened attention during their preparation. In addition to the commonly observed symptoms of headaches, palpitations, and sweating, anesthesiologists should also carefully evaluate other signs, particularly neurological and metabolic symptoms, because the secretion of PCC is a neurological active substance, including NE, epinephrine (E), and dopamine [10]. For instance, tumors secreting NE are more likely to cause hypertension and heart rate changes, while those secreting dopamine present with more insidious symptoms [11]. Notably, psychological manifestations such as anxiety and panic affect approximately 20%–40% of patients, while around 40% experience hyperglycemia. Additionally, patients may present with comorbidities such as migraine, stroke, diencephalic epilepsy, meningioma, porphyria, and factitious disorders and receive corresponding drug treatments, which are actually caused by PCC [12]. In this case, despite the patient's denial of any related symptoms, postoperative monitoring uncovered that he had suffered from sporadic, mild, and transient discomfort in the occipital region and bouts of anxiety over the preceding year. These symptoms were alleviated post‐surgery. Consequently, even minor and infrequent psychological manifestations warrant considerable attention from anesthesiologists.

Second, while the assessment of CAs and their metabolites is a crucial step in diagnosing or ruling out the disease, their diagnostic utility is somewhat restricted due to the susceptibility of their plasma levels to various factors and their episodic secretion patterns [13]. The preferred laboratory approach involves measuring plasma‐free or urinary MN and normetanephrine (NMN), collectively known as MNs. Owing to their longer half‐life and greater stability, MNs offer superior sensitivity and specificity compared to CAs and VMAs, making them widely recommended for the diagnosis of PPGLs [14, 15]. Additionally, although VMA, being the terminal metabolite of NE and E, has lower sensitivity (ranging from 46% to 77%), it boasts higher specificity (between 86% and 99%) and can serve as a complementary measure to CA and MNs testing [13]. The detection of 3‐methoxytyramine (3‐MT), an intermediate metabolite of dopamine, aids in enhancing the sensitivity of screening for head and neck PGLs and is of significant value in diagnosing metastatic tumors [16].

While conventional imaging modalities such as computed tomography (CT) and magnetic resonance imaging (MRI) may face challenges in distinguishing asymptomatic and small‐volume tumors like PCC [17, 18], emerging imaging techniques offer enhanced specificity and sensitivity for the diagnosis of PCC. For instance, metaiodobenzylguanidine (MIBG) scintigraphy and positron emission tomography (PET), particularly 18F‐FDOPA PET, demonstrate remarkable potential. As a fluorinated analog of L‐DOPA, 18F‐FDOPA can selectively enter neuroendocrine cells via the amino acid transporter (LAT1/CD98), exhibiting exceptional specificity for PPGL (95%–100%), surpassing other radiotracers [19, 20].

Furthermore, approximately 40% of these tumors are associated with genetic mutations that influence their biochemical characteristics and malignant potential. Therefore, genetic examination can offer valuable insights for functional diagnosis, as well as guide personalized treatment and prognosis evaluation [20, 21]. There are three primary genetic clusters based on the characteristics of PPGL. The pseudohypoxia signaling cluster (Cluster‐1) is correlated with the HIF pathway and is typically linked to more aggressive tumor behavior. The kinase signaling cluster (Cluster‐2) is associated with genes such as RET and NF1, which are related to higher tumor differentiation, lower malignant potential, and better prognosis. The Wnt signaling cluster (Cluster‐3), connected to the Wnt pathway, is usually sporadic and characterized by invasiveness and metastatic potential [22].

Considering the high cost and certain invasiveness of the above technologies, Georgiana Constantinescu et al. indicate that it is essential to implement blood pressure control, prevention of tachycardia, and volume expansion in all patients, even those pre‐diagnosed with nonfunctioning adrenal adenoma. Preoperatively, α‐adrenergic receptor antagonists, such as phenoxybenzamine are recommended for 14 days, starting at 10 mg once or twice daily and gradually increasing to 20–100 mg/day to control hypertension and stabilize vascular tone. Selective α_1_‐blockers like doxazosin may also be used to reduce the risk of postoperative hypotension [23, 24]. Once adequate α‐blockade is achieved, β‐blockers such as propranolol (10 mg every 6 h) can be introduced to manage tachycardia, ensuring they are started only after α‐blockade to prevent hypertensive crises. Calcium channel blockers may be added if tachycardia persists despite α‐ and β‐blockade [24]. Additionally, initiating a high‐sodium diet (> 5000 mg/day) on the 2nd to 3rd day of α‐adrenergic receptor blocker therapy aids in vascular volume expansion and reduces orthostatic hypotension, though it should be used cautiously in patients with congestive heart failure or renal insufficiency [25]. In our center, patients undergoing benign adrenal tumor resection routinely receive preoperative volume expansion for 3 days (3000 mL/day). As demonstrated in this case, the intraoperative hypotension was easily reversed even without completed fluid resuscitation and preoperative pharmacological intervention, further emphasizing the crucial role of short‐term volume management in mitigating potential fatal intraoperative hemodynamic fluctuations.

### 3.2 Intraoperative Monitoring and Intervention

Invasive arterial blood pressure and BIS monitoring were recommended perioperatively to ensure real‐time monitoring of vital signs, enabling the team to promptly respond to sudden elevations in blood pressure and drops in heart rate, thereby preventing further cardiovascular events. Notably, changes in heart rate may be an earlier warning signal of NE‐type PCC and should be given high attention. The choice of anesthetic agents should avoid cardiotoxic drugs; sevoflurane is recommended as the preferred inhalational anesthetic due to its lower risk of arrhythmias and minimal cardiovascular suppression. Muscle relaxants such as rocuronium, vecuronium, and cisatracurium are preferred for their minimal effects on the autonomic nervous system and lower likelihood of histamine release. Fentanyl is favored as a safe short‐acting analgesic, whereas morphine should be avoided to reduce the risk of histamine‐mediated side effects [26]. Additionally, the anesthesia team preemptively prepared vasoactive medications for rapid pharmacological intervention, such as urapidil, nicardipine, esmolol, and NE, as well as emergency drugs like atropine. Among them, urapidil and esmolol can effectively control sudden elevations in blood pressure and tachycardia caused by CA, while NE is used to manage hypotension occurring after tumor resection [27]. In this case, when the surgeon first manipulated the adrenal tumor, the patient's blood pressure surged within approximately 1 min, accompanied by a significant drop in heart rate. This pattern suggested NE secretion by the tumor, leading to α‐receptor activation, vasoconstriction, and a subsequent blood pressure spike, while the baroreceptor reflex induced bradycardia [10]. Faced with the risk of hypertensive crisis and bradycardia, it was crucial to quickly mitigate organ damage and elevate heart rate. We used the α‐receptor antagonist urapidil to lower blood pressure and atropine to counteract vagal effects, stabilizing hemodynamics rapidly. This approach's advantage is its swift action, essential for managing acute fluctuations. However, precise dosing is vital to prevent overcorrection.

Key measures to prevent excessive CA release include minimizing tumor manipulation and early ligation of the adrenal vein [28]. Following the ligation of the adrenal vein, attention must be redirected toward managing the physiological changes associated with a rapid decrease in serum CA levels, such as vasodilation and hypotension. Moreover, long‐term exposure to high CA could inhibit insulin secretion, along with surgical stress responses and fasting, which significantly increase the incidence of hypoglycemia following tumor removal. Electrolyte disorders may be secondary to the large intraoperative fluid infusion, blood loss, and hormonal level changes. Actually, a recent study involving 10 hospitals and 159 PCC patients reported a postoperative complication rate of 19% after PCC resection, mainly including hypotension, hypoglycemia, and metabolic and electrolyte disorders [29]. Therefore, postoperative blood pressure, blood glucose, and electrolyte levels should be closely monitored. In this case, although no significant metabolic and electrolyte disorders were observed, postoperative blood gas analysis and electrolyte monitoring should still be emphasized to detect and manage potential problems promptly.

### 3.3 Pathogenesis and Prognosis

The pathogenesis of silent PCC is complex and important for clinical presentation and prognosis. Though it is thought the bigger tumor (> 5 cm) is relatively symptomatic, there is a lack of a simple linear relationship between tumor size and symptoms, because it is also regulated by receptor density and sensitivity [30, 31]. In this case, immunohistochemical results showed positivity for CgA and Syn, indicating a neuroendocrine nature, while S‐100 positive sustentacular cells suggested a lower risk of malignancy. Taken together, the results supported the diagnosis of PCC. Tumor cells were polygonal or round with abundant eosinophilic cytoplasm, and cells were separated by fine capillaries into the typical Zellballen pattern, which is significant as it is a characteristic histological feature of PCCs, aiding in their identification and confirmation of their neuroendocrine origin. Consistent with other PGL case reports [32, 33, 34], the Zellballen pattern was also observed in this case.

The recurrence rate after PCC surgery ranges from 14% to 30% and is particularly higher in patients with genetic mutations [35, 36]. A Ki‐67 index greater than 3% or SDHB negativity is usually associated with a higher risk of recurrence and metastasis [37]. Here, reticulin staining showed destruction of acinar structures, suggesting possible local invasiveness; however, the 2% Ki‐67 proliferation index indicates low proliferative activity and a favorable prognosis. The prognosis after PCC surgery is generally favorable, especially when the tumor is completely resected and lacks malignant features. Even so, regular follow‐up is crucial.

## 4 Conclusion

We present a perioperative management approach for a scheduled general anesthesia in a patient with silent PCC, emphasizing the key considerations for these easily overlooked yet potentially fatal conditions. This case underscores the significance of comprehensive preoperative evaluation, even in the absence of classical symptoms, and highlights the imperative to maintain vigilance throughout surgery. Early identification and meticulous perioperative planning are crucial in preventing severe complications and optimizing patient outcomes.

## Author Contributions

**Kai Lin:** investigation, writing – original draft, writing – review and editing. **Xuan Li:** writing – original draft. **Tian Guo:** writing – original draft. **Haixing Zhong:** investigation, supervision, writing – review and editing.

## Consent

Written informed consent was obtained from the patient for publication of this case report.

## Conflicts of Interest

The authors declare no conflicts of interest.

## Data Availability

The authors have nothing to report.
